# Vagus Nerve Stimulation-Induced Laryngeal Motor Evoked Potentials: A Possible Biomarker of Effective Nerve Activation

**DOI:** 10.3389/fnins.2019.00880

**Published:** 2019-08-27

**Authors:** Simone Vespa, Lars Stumpp, Charlotte Bouckaert, Jean Delbeke, Hugo Smets, Joaquin Cury, Susana Ferrao Santos, Herbert Rooijakkers, Antoine Nonclercq, Robrecht Raedt, Kristl Vonck, Riëm El Tahry

**Affiliations:** ^1^Institute of Neuroscience, Université catholique de Louvain, Brussels, Belgium; ^2^4Brain, Institute for Neurosciences, Ghent University, Ghent, Belgium; ^3^Bio, Electro And Mechanical Systems, Université Libre de Bruxelles, Brussels, Belgium; ^4^Centre for Refractory Epilepsy, Cliniques Universitaires Saint-Luc, Brussels, Belgium; ^5^Department of Neurosurgery, Cliniques Universitaires Saint-Luc, Brussels, Belgium; ^6^Reference Center for Refractory Epilepsy, Department of Neurology, Ghent University, Ghent, Belgium

**Keywords:** vagus nerve stimulation (VNS), epilepsy, biomarkers, larynx, motor evoked potentials, internal consistency, reliability, electrical axis

## Abstract

Vagus nerve stimulation (VNS) therapy is associated with laryngeal muscle activation and induces voice modifications, well-known side effects of the therapy resulting from co-activation of the recurrent laryngeal nerve. In this study, we describe the non-invasive transcutaneous recording of laryngeal motor evoked potentials (LMEPs), which could serve as a biomarker of effective nerve activation and individual titration in patients with drug-resistant epilepsy. We recruited drug-resistant epileptic patients treated for at least 6 months with a VNS. Trains of 600–1200 VNS pulses were delivered with increasing current outputs. We placed six skin electrodes on the ventral surface of the neck, in order to record LMEPs whenever the laryngeal muscular threshold was reached. We studied the internal consistency and the variability of LMEP recordings, and compared different methods for amplitude calculation. Recruitment curves were built based on the stimulus–response relationship. We also determined the electrical axis of the LMEPs dipole in order to define the optimal electrode placement for LMEPs recording in a clinical setting. LMEPs were successfully recorded in 11/11 patients. The LMEPs threshold ranged from 0.25 to 1 mA (median 0.50 mA), and onset latency was between 5.37 and 8.77 ms. The signal-to-noise ratio was outstanding in 10/11 patients. In these cases, excellent reliability (Intraclass correlation coefficient, ICC > 0.90 across three different amplitude measurements) was achieved with 10 sample averages. Moreover, our recordings showed very good internal consistency (Cronbach’s alpha > 0.95 for 10 epochs). Area-under-the-curve and peak-to-peak measurement proved to be complementary methods for amplitude calculation. Finally, we determined that an optimal derivation requires only two recording electrodes, aligned on a horizontal axis around the laryngeal prominence. In conclusion, we describe here an optimal methodology for the recording of VNS-induced motor evoked responses from the larynx. Although further clinical validation is still necessary, LMEPs might be useful as a non-invasive marker of effective nerve activation, and as an aid for the clinician to perform a more rational titration of VNS parameters.

## Introduction

Epilepsy is considered ‘drug-resistant’ when the patient is not seizure free despite treatment with at least two anti-epileptic drugs at correct dosages (Kwan et al., 2010). In such cases, patients are referred to a specialized epilepsy center for pre-surgical evaluation. When surgery is not indicated, for example in the case of generalized epilepsy, or of a non-localizable or multifocal seizure onset, neuromodulation by vagus nerve stimulation (VNS) may be an effective alternative. VNS was developed in the ‘80s and consists of an implanted device that generates and delivers electrical pulses to the left cervical vagus nerve by means of helical electrodes. The efficacy of VNS has been confirmed by randomized controlled trials (Ben-Menachem et al., 1994; Handforth et al., 1998) and meta-analysis (Englot et al., 2011). Despite the demonstrated clinical efficacy, one third of patients still do not benefit from the therapy and are classified as ‘non-responders’ (Boon et al., 2002). The response to VNS is variable from patient to patient, both in terms of efficacy and of side effects (Vonck et al., 2004). The mechanisms of action of VNS remain poorly understood (Krahl and Clark, 2012) and there is a lack of established predictors of efficacy applicable to individual subjects (Ghaemi et al., 2010; Englot et al., 2016). One of the possibilities to improve the therapy would be to characterize a biomarker for effective vagal nerve fiber activation, which is a prerequisite for therapeutic efficacy. An objective biomarker could aid neurologists to titrate the delivered electrical current (defined by the stimulation parameters) in a more rational and personalized manner. Such an attempt was made by recording cortical potentials evoked intraoperatively by the VNS (Usami et al., 2013). These authors used muscle relaxants to demonstrate that the early components of their responses were linked to afferent vagal signals, whereas late components were related to VNS-induced motor activity.

In the hope to develop a non-invasive alternative that would be more applicable in clinical routine, we explored the muscular effects of the vagal stimulation. VNS therapy co-activates the recurrent laryngeal nerve, which branches off from the vagus nerve at the level of the aortic arch, below the standard location of the implanted electrode. The recurrent laryngeal nerve carries low threshold vagal Aα motor fibers innervating all the laryngeal muscles (except the cricothyroid) and part of the pharynx (Krahl, 2012). Among the side effects reported by patients implanted with VNS, voice alteration during stimulation periods affects 20 to 62% of them (DeGiorgio et al., 2000; Zalvan et al., 2003; Englot et al., 2011). These symptoms are explained by an irregular contraction of antagonist intrinsic laryngeal muscles (Kersing et al., 2002). VNS-induced activation of the recurrent laryngeal nerve results in laryngeal motor evoked potentials (LMEPs), which were previously successfully recorded intraoperatively in animals with implanted cervical vagus nerve electrodes (El Tahry et al., 2011; Mollet et al., 2013; Grimonprez et al., 2015). Nerve lesions and blocking of neuromuscular junction at laryngeal level confirmed the motor origin of the recorded responses. Animal evidence on LMEPs was later translated into a clinical setting, and LMEPs were specifically recorded in epileptic patients through surface cervical electrodes, in different orientations around the larynx (Bouckaert et al., 2018).

The present study is a feasibility study with the aim of confirming the possibility of non-invasive recording of LMEPs in patients treated with VNS therapy. The primary goal of this study is to establish the optimal clinical routine methodology for surface recording of human VNS-induced LMEPs, and to extract their most pertinent features. We describe the general characteristics of LMEP recordings, including onset latency, amplitude, and variability as well as individual recruitment (stimulus–response) curves. In addition, we explored the optimal bipolar electrode orientation and sought the most reliable LMEP amplitude quantification method.

## Materials and Methods

### Patient Selection

Drug-resistant epileptic patients treated with VNS therapy were recruited from the VNS follow-up database of the Centre for Refractory Epilepsy of Saint Luc University Hospital, Brussels, Belgium. The Ethics Committee of Saint-Luc Hospital approved the study procedures (Reference No. 2018/07NOV/416). Informed consent to the study was given prior to any investigation. Inclusion criteria were: (i) patient aged between 18 and 65 years; (ii) implanted cervical VNS device (DemiPulse^®^ Model 103 or DemiPulse Duo^®^ Model 104 or AspireHC^®^ Model 105 or AspireSR^®^ Model 106; LivaNova, Inc., London, United Kingdom) for at least 6 months; (iii) VNS electrode impedance between 2 and 5 kOhm, as assessed on the day of the investigation. Exclusion criteria were: (i) presence of a concomitant laryngeal pathology or recurrent laryngeal nerve damage, independent from VNS; (ii) important VNS side effects reported by the patient, such as severe dyspnea (grade III–IV) or severe pain in the neck/ear region. Intermittent hoarseness was allowed. The clinical response to VNS was determined based on the clinical report of the latest available follow-up visit. Patients were considered as responders to VNS if a reduction ≥ 50% in seizure frequency was obtained. If the reduction in seizure frequency was < 50%, patients were considered as non-responders.

### Techniques

**FIGURE 1:**
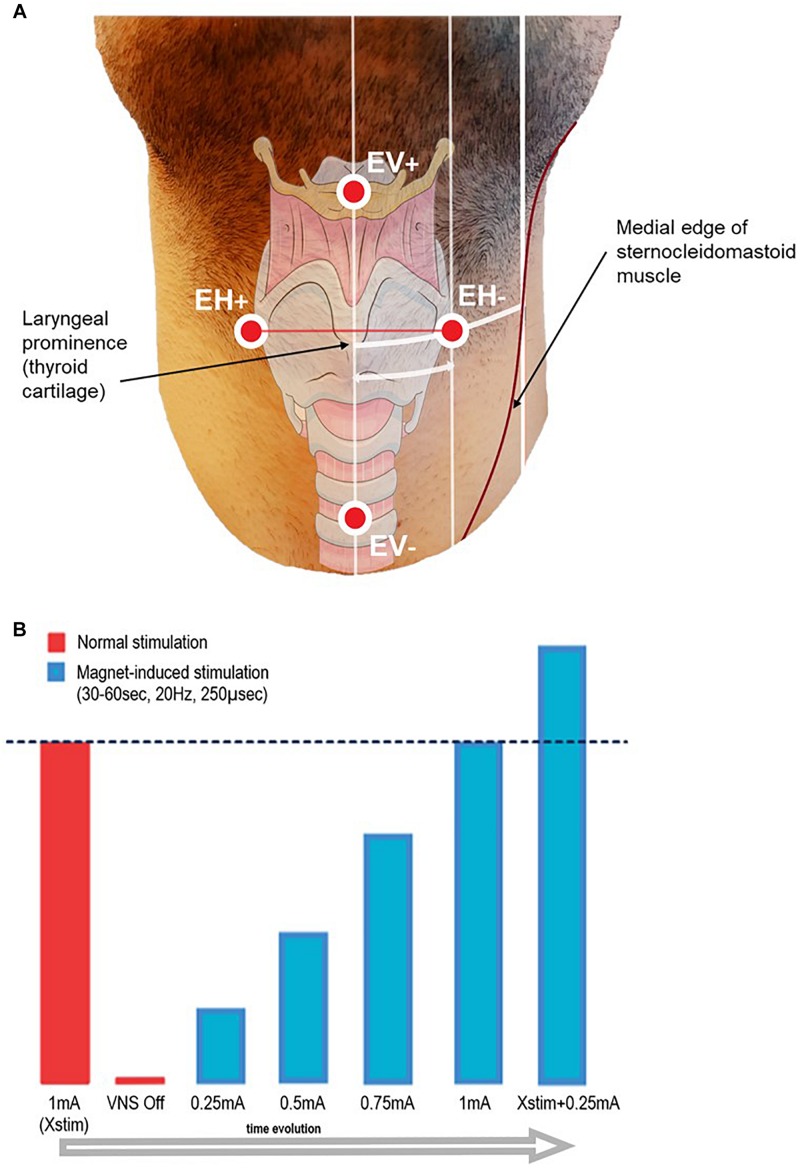
**(A)** Schematic representation of the recording surface electrode placement. EH+/EH– electrodes were aligned horizontally, and placed symmetrically on each side of the laryngeal prominence, at half the distance between the midline and the medial edge of the sternocleidomastoid muscle (approximately 4 cm). EV+/EV– electrodes were aligned vertically, placed on the midline 6 cm above and below the laryngeal prominence. Additionally, two electrodes for artifact recording (Art1–Art2) were placed on the left mastoid and below the left clavicle side of the neck (not shown in figure), and one grounding electrode was placed on the forehead (not shown in figure). The distances take into account an approximation due to the curvature of the neck and due to the perspective of the image. **(B)** Scheme of the stimulation protocol.

The experiments were performed at the Neurophysiology Unit of Saint Luc University Hospital, Brussels, Belgium. Evoked potentials (EP) were recorded using an EEG/EP digital acquisition system (Matrix 1005, Micromed, Mogliano Veneto, Italy). Sampling rate was set at 32 kHz (except for one case sampled at 2048 Hz), with a voltage window of ± 200 μV. High-pass filtering was 0.15 Hz (time constant = 1 s). Low-pass cut-off was 8 kHz for cases sampled at 32 kHz, and 540 Hz for the case sampled at 2048 Hz. The impedance of the VNS lead electrode was checked by means of the standard handheld programing wand before starting the procedure. If an impedance > 5 kOhm was measured, the patient was excluded from the study. Seven surface electrodes (Skintact FS-50 Gel ECG Electrodes) were placed on the skin of the patient after local skin scrubbing with Weaver Prep Gel. On the ventral skin surface of the patient’s neck, four electrodes were arranged in two bipolar derivations: one vertical (EV−/EV+) and one horizontal (EH−/EH+). The EV−/EV+ electrodes were placed 6 cm above and below the laryngeal prominence, respectively; EH−/EH+ were placed symmetrically and horizontally to the right and to the left of the same reference point, at half the distance between the laryngeal prominence and the medial edge of the sternocleidomastoid muscle, i.e., about 4 cm laterally to the midline (see Figure 1). Two additional electrodes (Art1/Art2) were placed laterally on the left mastoid and below the middle part of the left clavicle respectively, forming a recording channel in-line with the stimulation artifact. Grounding was insured by a forehead electrode. After connection to the acquisition system, skin electrode impedances were checked to be < 20 kOhm.

### Stimulation Protocol

At onset, patients were asked to minimize neck movements and to refrain from speaking. Therapeutic VNS (‘Normal stimulation’) was temporarily switched off by setting the current output to 0 mA. A 2-min baseline recording was acquired before LMEP elicitation in order to allow high pass filter stabilization. VNS was activated on-demand by sweeping an external magnet over the implanted stimulator, while the recording was running. The starting current output was set at 0.25 mA, and then was gradually ramped up by steps of 0.25 mA at each magnet sweep. Based on the individual patient’s therapeutic current output (Xstim), we stimulated until a value of Xstim + 0.25 mA was reached (see Figure 1). In order to ensure similar conditions across patients, pulse width and frequency were left unchanged at respectively 250 μs and 20 Hz. Each magnet sweep was programed to produce a 30 or 60 s train of stimulation (VNS On time according to the patient’s tolerability) each yielding 600 or 1200 VNS pulses per current output level.

### Data Analysis

Signals were analyzed offline using MATLAB 2018a software (MathWorks, Natick, MA, United States). Automatic trigger detection was performed on MATLAB, with the zero reference time corresponding to the first negative flank of the VNS stimulation artifact at electrode EH−. A 50 Hz notch filter was applied to the electromyographic recording, which was thereafter segmented in samples from −3 to + 25 ms around the corresponding stimulation artifact. Hereafter, we thus refer to these individual 28 ms samples as ‘epochs.’ The pre-processing ended with a baseline correction. All epochs obtained at the same current output were overlaid and visually screened for outliers, e.g., epochs from segments affected by major artifacts (mostly coughing, phonation, or other patient movement). Due to a “ramp-up” and “ramp-down” of the current output during the first and last 2 s of stimulation trains, epochs not corresponding to the pulses at target current intensity were also excluded.

**FIGURE 2:**
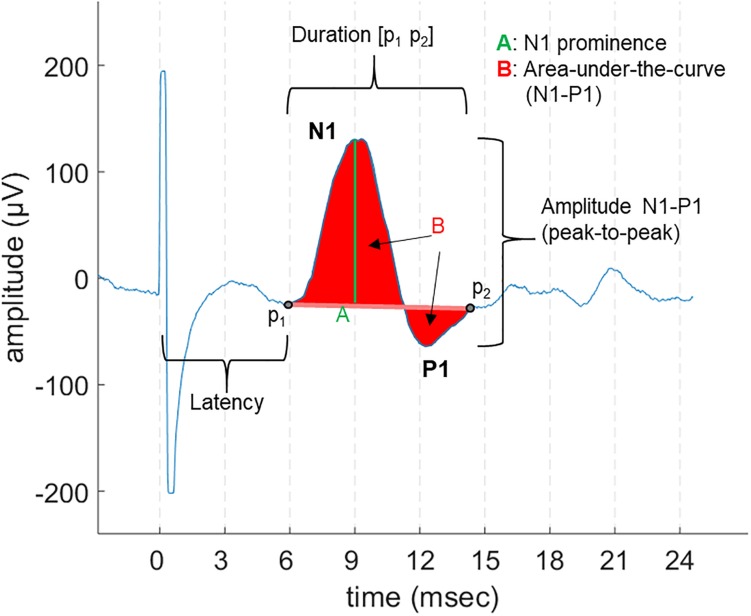
An example of LMEP and its characteristics, as displayed in a 28 ms epoch. The different measurements for amplitude that were are studied are indicated: area-under-the-curve (AUC), peak-to-peak (P2P), and N1 prominence.

Laryngeal motor evoked potentials were defined as the reproducible bi- or tri-phasic waveforms appearing as an all-or-nothing response to the stimulation, with an expected onset latency range of 5–9 ms, as described in previous invasive laryngeal EMG studies (Ardesch et al., 2010). For each patient, we first determined the threshold, i.e., the lowest current output (a multiple of 0.25 mA) sufficient to evoke LMEPs visible either in single traces or after averaging. We determined the following LMEP characteristics (Figure 2):

- Latency: the time between the negative peak of the stimulation artifact and the initial negative (at EH−) deflection of LMEP (t_0_–p_1_).
- Duration: the time between the first LMEP negative deflection (p_1_) and the return to baseline (p_2_).
- Peak-to-peak (P2P) amplitude: the peak-to-peak difference between the initial N1 peak and the following P1 peak.
- Area-under-the-curve (AUC) amplitude: the integral of N1 and P1 waves, all referred to the p_1_-p_2_ line as zero level.
- Prominence amplitude (N1 prominence): the amplitude of N1 peak over the p_1_ amplitude value, taken as zero level.

#### Amplitude Measurements

Different methods can be applied to quantify the amplitude of a motor EP, based on either peak sizes or signal integration (area) measurements. Different metrics are widely accepted (Lavoie et al., 1995; Cacchio et al., 2009; Joyeux et al., 2017). We compared the results of the LMEP amplitude evaluation using a P2P metric, a single peak amplitude (N1 prominence) and the integral AUC measurements.

For each measure, we calculated the mean values per train of stimulation based on the *grand average* (average over the maximum number of epochs available in a train). We first measured the mutual agreement of the three different amplitude measurements, as an indication of their inter-exchangeability and of the overall reliability of EP measures (Joyeux et al., 2017). We calculated the ICC(3,k) for inter-rater reliability, in which each method is considered as a different rater, and their consistency across trials is tested (McGraw and Wong, 1996; Koo and Li, 2016; Joyeux et al., 2017). Values of ICC > 0.75 are considered as an indicator of good reliability, and values > 0.90 of excellent reliability (Koo and Li, 2016). In addition, we measured the variability within one train of stimulation for each method and calculated their relative coefficient of variation (CV) = %(SD/mean values) per train. CV can provide information on whether the measurement is more or less sensitive to intra-train fluctuations, such as respiration, but also provides an indirect estimate of the signal-to-noise ratio (SNR) – the lower the CV, the higher the SNR (Okamoto et al., 2015).

#### Internal Consistency Analysis: Cronbach’s Alpha and Pearson’s Correlation Coefficient

Based on the recording hardware, on the nature of the quantification method and on the SNR, EP single epoch recordings may have lower or higher internal consistency across observations (Fabiani et al., 1987; Thigpen et al., 2017). The estimation of internal consistency is thus crucial in defining how many epochs should be averaged for the EP characteristics measurements to yield reliable results (Cohen and Polich, 1997; Olvet and Hajcak, 2009; Goldsworthy et al., 2016). Internal consistency can be practically evaluated using Cronbach’s alpha, which characterizes the similarity between repeated measurements by correlating all possible subsets of the measurements (Cronbach and Warrington, 1951; Fabiani et al., 1987; Thigpen et al., 2017). Values > 0.9 indicate excellent internal consistency (Hinton et al., 2014).

In the present study, we investigated the internal consistency of LMEPs, using Cronbach’s alpha and Pearson’s correlation coefficient, with a double purpose: (i) to define a minimal number of epochs to be included in each average in order to yield consistent measurements; (ii) to identify the response amplitude metrics showing the highest internal consistency (see section “Amplitude Measurements”). We analyzed the values of LMEP amplitude across increasing numbers of epochs (5, 10, 20, 50, 100, 200) selected randomly from a same train. First, matrices for Cronbach’s alpha calculation were built with single-epoch amplitude value in a given metric, while the different patients accounted for different observations (Fabiani et al., 1987; Thigpen et al., 2017). Alpha values were obtained for each number of epochs, and for each amplitude metric.

In addition, in order to assess the effect of the number of epochs on the final results, we compared the correlation of *small-number averages* (i.e., obtained with 5, 10, 20, 50, 100, 200 epochs) with the grand average. This was quantified on the basis of a Pearson correlation coefficient (Olvet and Hajcak, 2009; Rietdijk et al., 2014).

#### Recruitment Curves

We aimed to fit a Boltzmann sigmoid function to the recruitment curves (Devanne et al., 1997), in order to describe the LMEP amplitudes (in μV) according to the following function of the stimulus current (in mA):

(1) $$L\,M\,E\,P\,a\,m\,p\,l\,i\,t\,u\,d\,e=\frac{L\,M\,E\,P_{\max}}{\left[1+\exp\left(\left(I_{50}-I\right)/k\right)\right]}$$

where *LMEPmax* is the maximum value reached by LMEP amplitude, *I*_50_ is the stimulus current value at which the response amplitude reaches half LMEPmax and *k* is the slope of the curve. Only curves with one current value below threshold, two at the level of *LMEPmax* (indicating saturation) and two on the ascending part of the slope, were considered for fitting with the equation above. Goodness of fit was reported in terms of *r*^2^ values (good fit = *r*^2^ > 0.95) and root mean square error (RMSE).

#### Electrical Axis

Only laryngeal muscles ipsilateral to the stimulated left vagus nerve are expected to be activated (Kersing et al., 2002; Ardesch et al., 2010). Therefore, despite the various orientations of these muscles, the resulting potential vector was expected to be asymmetrical between the left and the right side. We aimed to determine the amplitude and direction of the maximal LMEP electrical axis, considering the two orthogonal derivations in the frontal plan as given by the EH and EV channels (Figure 1). The horizontal vector to the right (EH+ electrode) is taken as (0°), while the value of 90°corresponds to the upwards vertical direction (EV+). The A_LMEP_ (Dipole vector amplitude) and D_LMEP_ (Dipole vector orientation) were calculated according to the equations:

(2) $$A_{L\,M\,E\,P}=S\,Q\,R\,T\,\left(E\,V\,{\left(t\,M\,a\,x\right)}^{2}+E\,H\,{\left(t\,M\,a\,x\right)}^{2}\right)$$

(3) $$D_{L\,M\,E\,P}=\pm \arctan\left(\frac{E\,V\,\left(t\,M\,a\,x\right)}{E\,H\,\left(t\,M\,a\,x\right)}\right)$$

where EV(tMax) and EH(tMax) are the amplitude in the vertical and horizontal derivation respectively at the time in which the sum of the latter two amplitudes reaches the highest value.

#### Electrodes Placement

In one patient, we tested how the distance of the electrodes from the midline could affect the qualitative and quantitative aspects of the recordings (SNR, amplitude of signals). To do so, we placed the electrodes laterally to the laryngeal prominence at three different distances: 2 cm, half distance to the medial edge of the sternocleidomastoid muscle as used in the rest of the recordings (i.e., about 4 cm) and close to the medial edge of the sternocleidomastoid muscle (i.e., about 6 cm) (Figure 1), and compared the obtained results. In the patient where this test was performed, no EV electrodes were placed.

### Statistical Analysis

Statistical analyses were performed using IBM SPSS Statistics for Windows, version 2.0 (IBM, Corp., Armonk, NY, United States). Mean values, standard deviation (SD) and 95% confidence intervals (C.I.) are reported unless stated otherwise. One-way ANOVA was used to assess whether significant differences existed between CV values across the three amplitude measurements. Statistical significance was considered at *p* < 0.05. More specific analyses, such as reliability statistics, are described above in the text.

## Results

Laryngeal motor evoked potentials were successfully recorded in all 11 patients (eight females, three males; mean age: 35.7 years). The mean time between implantation and the present testing was 67.9 months (range: 8–169 months). 3/11 patients were classified as responders to VNS therapy, while 8/11 as non-responders. A detailed list of patient records is provided in Table 1. In 9/11 patients, LMEPs were predominantly visible in the EH compared to the EV channel. In 2/11 patients (PAT 8, PAT 9) LMEPs appeared with comparable amplitudes in both EH and EV channels. The stimulation artifact was equally visible in all three derivations (EV, EH, and Art), and appeared with an initial negative peak in reference to EH− electrode. Hereafter, all reported results refer to recordings from the EH channel except for analysis of the dipole orientation.

**TABLE 1:** Clinical records of the study population.

		**Duration**		**Pulse**		**Clinical**	
	**Age**	**of VNS**		**generator**		**response**	**VNS clinical**
**PAT no.**	**(range)**	**implant**	**VNS normal parameters**	**model**	**Epilepsy type**	**to VNS**	**side effects**
PAT 1	26–30	4y 10m	1.75 mA; 20 Hz; 250 usec; 30 s On 3 min Off	106	Focal epilepsy, unknown etiology	NR	NA
PAT 2	21–25	8m	1 mA; 30 Hz; 500 usec; 30 s ON 5 min Off	106	Focal epilepsy, unknown etiology	R	Hoarseness
PAT 3	61–65	8y 3m	1.5 mA; 20 Hz; 250 usec; 14 s On 1.1 min Off	103	Focal epilepsy, structural etiology	NR	NA
PAT 4	31–35	6y 2m	1.75 mA; 25 Hz; 250 usec; 30 s On 5 min Off	103	Focal epilepsy, infectious etiology	NR	NA
PAT 5	31–35	8y 1m	2 mA; 25 Hz; 250 usec; 30 s On 1.1 min Off	106	Focal epilepsy, genetic etiology	NR	NA
PAT 6	18–20	2y 2m	1.25 mA; 20 Hz; 250 usec; 30 s On 5 min off	106	Focal epilepsy, unknown etiology	NR	NA
PAT 7	26–30	4y	1 mA; 20 Hz; 250 usec; 30 s On 5 min Off	103	Generalized epilepsy,	R	NA
PAT 8	51–55	14y 1m	1 mA; 30 Hz; 500 usec; 30 s On 5 min Off	103	Focal epilepsy, structural etiology	NR	NA
PAT 9	51–55	5y 8m	1 mA; 30 Hz; 500 usec; 30 s On 5 min Off	106	Focal epilepsy, structural etiology	R	Transient neck pain
PAT 10	18–20	4y 5m	1.75 mA; 30 Hz; 500 usec; 30 s On 5 min Off	103	Focal epilepsy, unknown etiology	NR	NA
PAT 11	41–45	3y 11m	1.75 mA; 20 Hz; 500 usec; 30 s On 5 min Off	103	Focal epilepsy, unknown etiology	NR	Hoarseness

*R, responder (**≥**50% seizure frequency reduction). NR, non-responder (<50% seizure frequency reduction).*

**FIGURE 3:**
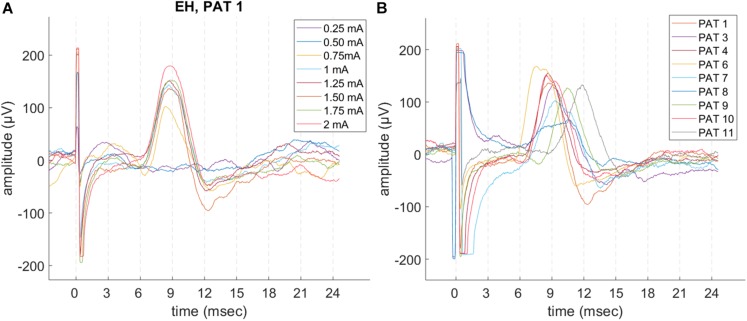
Example of LMEP single epochs, in patients with a good signal-to-noise ratio (SNR) and sampling rate at 32 kHz. **(A)** Intra-patient reproducibility (PAT 1), in different trains at increasing current output. **(B)** LMEPs recorded across different patients, at their respective normal current output (1–1.75 mA). Variation of amplitudes and latencies can be appreciated. Latencies are comprised in a 5.4–8.7 ms range (mean: 6.89 ms). PAT 2 is not shown due to a sampling rate of 2048 Hz that hampers a meaningful overlay. PAT 5 is not shown due to low SNR.

Laryngeal motor evoked potentials were highly reproducible at intra and inter-patient level (Figure 3 and Table 2). For each recorded train of pulses, 210 to 1200 valid epochs (mean = 893) were included in the grand average. Within a train, all LMEP characteristics (amplitudes, latency, and duration) showed a normal Gaussian distribution. LMEPs followed the stimulation artifact with a mean onset latency of 6.89 ms (range: 5.37–8.72 ms). The sampling rate at 2048 Hz (PAT 2) did not affect the main features of LMEPs. The mean duration of the responses was 10.6 ms (range: 8.03–14.65 ms).

**TABLE 2:** Laryngeal motor evoked potential (LMEP) characteristics at therapeutic current output (Xstim) and calculated based on the grand average.

	**Threshold**	**Latency**	**Duration**			
**PAT no.**	**(mA)**	**(ms)**	**(ms)**	**P2P amplitude (μV)**	**AUC amplitude (μV x ms)**	**N1 prominence amplitude (μV)**
PAT 1	0.75	6.26	10.29	Mean = 204.8 ± 19.03	Mean = 17346 ± 1562.5	Mean = 157.74 ± 17.26
PAT 2	0.5	5.37	14.65	Mean = 178.15 ± 14.86	Mean = 11918 ± 101	Mean = 65.46 ± 14.56
PAT 3	0.75	7.11	9.24	Mean = 172.67 ± 24.32	Mean = 11703 ± 2049.5	Mean = 96.12 ± 14.09
PAT 4	0.25	6.41	12.05	Mean = 181.55 ± 16.29	Mean = 15570.7 ± 1323	Mean = 141.02 ± 10.53
PAT 5	1	8.33	11.26	Mean = 75.26 ± NA	Mean = 8180.3 ± NA	Mean = 66.69 ± NA
PAT 6	0.5	5.4	11.08	Mean = 238.35 ± 11.64	Mean 23300.3 ± 1044.6	Mean = 175.31 ± 13.6
PAT 7	0.5	6.93	8.15	Mean = 138.21 ± 15.83	Mean = 14164.7 ± 1834	Mean = 112.75 ± 14.91
PAT 8	0.75	6.81	8.03	Mean = 66.83 ± 10.94	Mean = 6612 ± 1063.7	Mean 52.37 ± 9.3
PAT 9	0.25	8.08	11.17	Mean = 169.92 ± 11.91	Mean = 14422.7 ± 1258.9	Mean = 136.05 ± 10.8
PAT 10	0.25	6.35	10.44	Mean = 205.74 ± 16.4	Mean = 19204.8 ± 17.63	Mean = 179.72 ± 15.91
PAT 11	0.5	8.72	10.22	Mean = 180.98 ± 5.33	Mean = 12923.5 ± 17.78	Mean = 140.74 ± 6.18

*PAT 5 measures were based on averaged parameters, therefore no SD was available.*

### Amplitude Measurements and Internal Consistency

Excellent mutual agreement was found across the three different amplitude measurement methods, with ICC = 0.943 (C.I. 0.806–0.988) for the grand average. The ICC was already > 0.9 when averaging 10 epochs, and it evolved only minimally when more epochs were added (Table 3).

**TABLE 3:** Values of inter-rater reliability (ICC) of the three methods (P2P, AUC, and N1 prominence) and internal reliability (Cronbach’s alpha and Pearson’s correlation coefficient between averages), calculated in patients with good signal-to-noise ratio (*n* = 10) at their normal current output.

		**5 epochs**	**10 epochs**	**20 epochs**	**50 epochs**	**100 epochs**	**200 epochs**
ICC		0.88 (0.60–0.97)	0.90 (0.67–0.98)	0.91 (0.70–0.98)	0.92 (0.74–0.98)	0.92 (0.73–0.98)	0.91 (0.70–0.98)
Peak-to-peak (P2P)	*Alpha*	0.907	0.962	0.984	0.993	0.996	0.998
	*Pearson*	0.926	0.955	0.954	0.960	0.975	0.974
Area-under-the-curve (AUC)	*Alpha*	0.979	0.990	0.994	0.997	0.999	0.999
	*Pearson*	0.988	0.991	0.989	0.991	0.993	0.996
N1 prominence (N1)	*Alpha*	0.959	0.978	0.991	0.996	0.998	0.999
	*Pearson*	0.968	0.981	0.981	0.986	0.996	0.996
							

The amplitude measurement with the lowest CV per train was obtained with the P2P method (mean: 10.90%, *SD:* 3.38%), followed by AUC (mean: 12.28%, *SD:* 3.99%) and N1 prominence (mean: 14.12%, *SD:* 4.36%). However, there was no significant difference between the mean values (one-way ANOVA test, *p* = 0.183).

**FIGURE 4:**
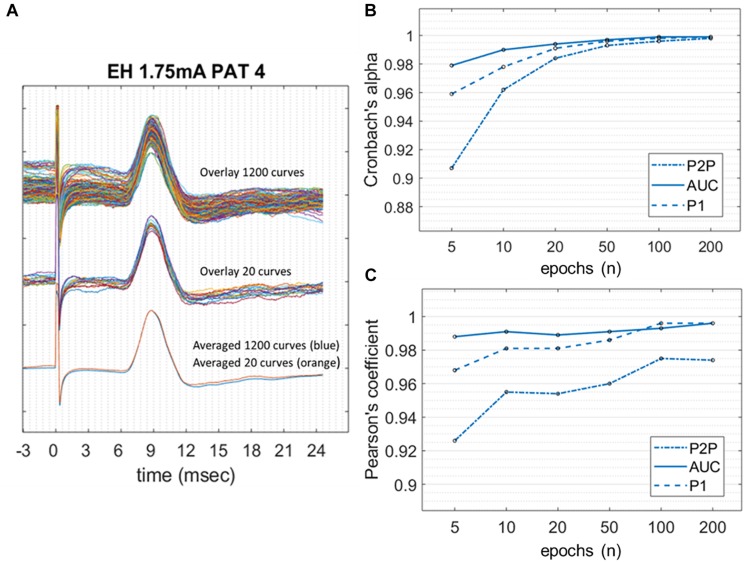
Internal consistency of LMEPs, in the case of good SNR. **(A)** LMEP recordings recorded from PAT 4. At the top of the figure, overlay of maximal amount of 1200 LMEPs. In the middle, overlay of randomly chosen 20 LMEPs. At the bottom, overlay of the averages from 1200 (blue line) and 20 curves (orange line), showing excellent robustness (Pearson’s coefficient > 0.95). **(B)** Values of Cronbach’s alpha for the peak-to-peak (P2P), area-under-the-curve (AUC) and N1 prominence amplitude calculation, at increasing number of curves included in the trial count. **(C)** Pearson’s correlation coefficient between LMEP amplitude results for small-number averages with those obtained at grand average.

Figure 4 shows the Cronbach’s alpha values calculated with increasing numbers of epochs in 10/11 patients, where a high SNR allowed measurement of amplitudes on single epochs. Considering all the three amplitude measurements, alpha values reached > 0.95 with 10 epochs averaged. Alpha values tend to a plateau (>0.99) when more than 50 epochs are included. Among the three methods, the AUC method showed the highest values of alpha at all numbers of epochs considered.

When calculating the Pearson correlation coefficient between the amplitude measurements of the small-number average and the grand average (Figure 4 and Table 3), AUC method showed the best correlation (>0.99 with 10 epoch average included). All three amplitude measurements yielded an excellent internal consistency from 10-epochs average on (Pearson’s coefficient > 0.95 for AUC, P2P, and N1 prominence). No substantial benefit was obtained if more than 100 epochs were included in the average.

**FIGURE 5:**
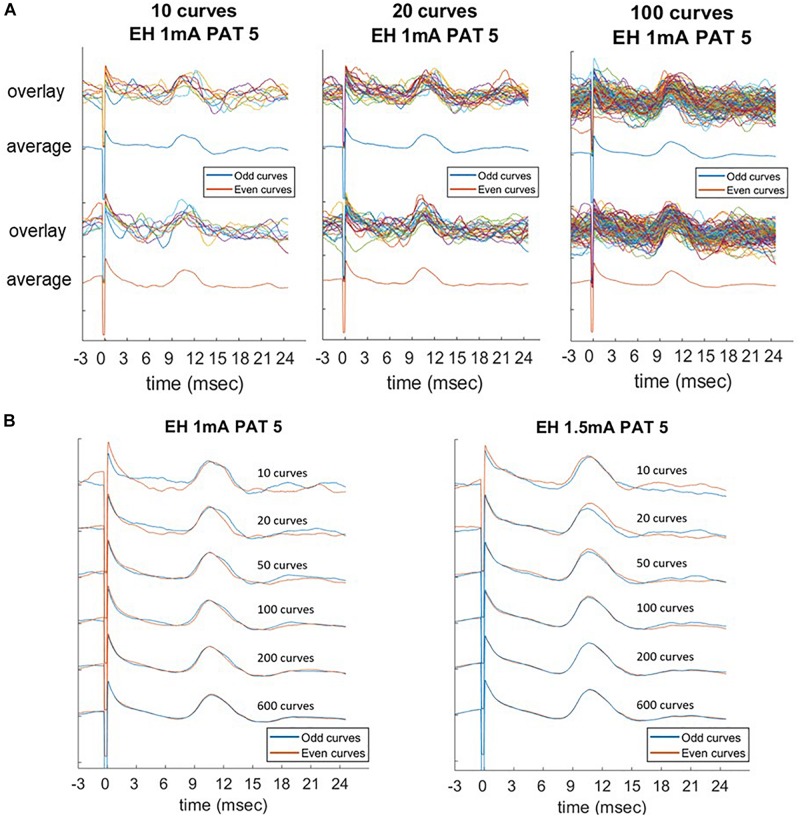
Laryngeal motor evoked potentials (LMEPs) recorded from PAT 5, which was the only patient with low signal-to-noise ratio. Comparison of average parameters at increasing number of epochs (10, 20, 50, 100, 200, maximum). **(A)** Consecutive odd-numbered single epochs were overlaid and averaged (blue color) and compared to consecutive even-numbered epochs (orange color). **(B)** Averaged curves were compared between them, with an increasing consistency of the curves across the two subgroups. Amplitude measurements become highly consistent as the number of epochs included in the average reaches 100 curves (Pearson correlation > 0.90).

Due to a low SNR, averaging was necessary to identify the LMEP in one single patient (PAT 5). In this case, only measurement values obtained from averaged epochs were available, thus precluding the calculation of a Cronbach’s alpha value. However, a slightly different method is available to assess the internal consistency of LMEP amplitude results, as a surrogate for the Cronbach’s alpha calculation (Green et al., 2016). We compared the amplitude values (P2P, AUC, N1 prominence considered altogether) from consecutive odd-numbered epochs with those from consecutive even-numbered epochs, for 10, 20, 50, 100, 200 and maximal number of epochs at 3 different current outputs (1, 1.5, 2 mA). As shown in Figure 5, averaged traces became stackable and the amplitudes consistent from 100 averaged epochs onwards (Pearson correlation coefficient > 0.90), while higher numbers did not significantly improve the traces Table 4.

**TABLE 4:** Odd-even reliability of small-number averages in PAT 5 (low signal-to-noise ratio) were calculated by Pearson’s correlation coefficient.

	**10 epochs**	**20 epochs**	**50 epochs**	**100 epochs**	**200 epochs**	**Grand**
Odd-even reliability	0.537	0.603	0.701	0.945	0.944	0.923

*The calculation was made taking into account the three different methods of amplitude calculation (peak-to-peak, area-under-the-curve, N1 prominence).*

### Recruitment Curves

**FIGURE 6:**
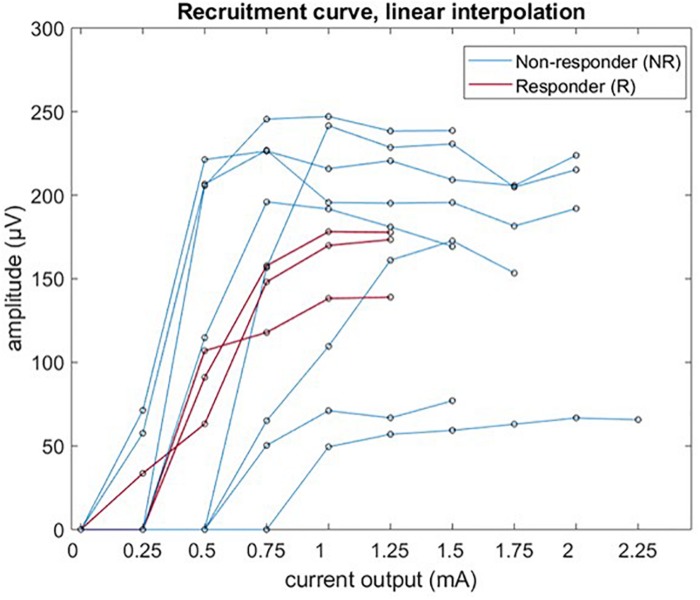
Recruitment curve of LMEP amplitude (peak-to-peak values) visualized by linear interpolation.

Laryngeal motor evoked potentials grand average P2P amplitude was plotted – with linear interpolation for the sake of clarity, as a function of the stimulation current for all 11 patients, and is shown in Figure 6. In only 3/11 cases there were enough data points available to fit a Boltzmann equation (values of *R*^2^ > 0.95). The calculated values of *I*_50_ were 0.50 mA (PAT 2), 0.86 mA (PAT 3), and 0.55 mA (PAT 9); values of *k* were 0.145, 0.148, and 0.082 μV/mA, respectively.

### Electrical Axis

**FIGURE 7:**
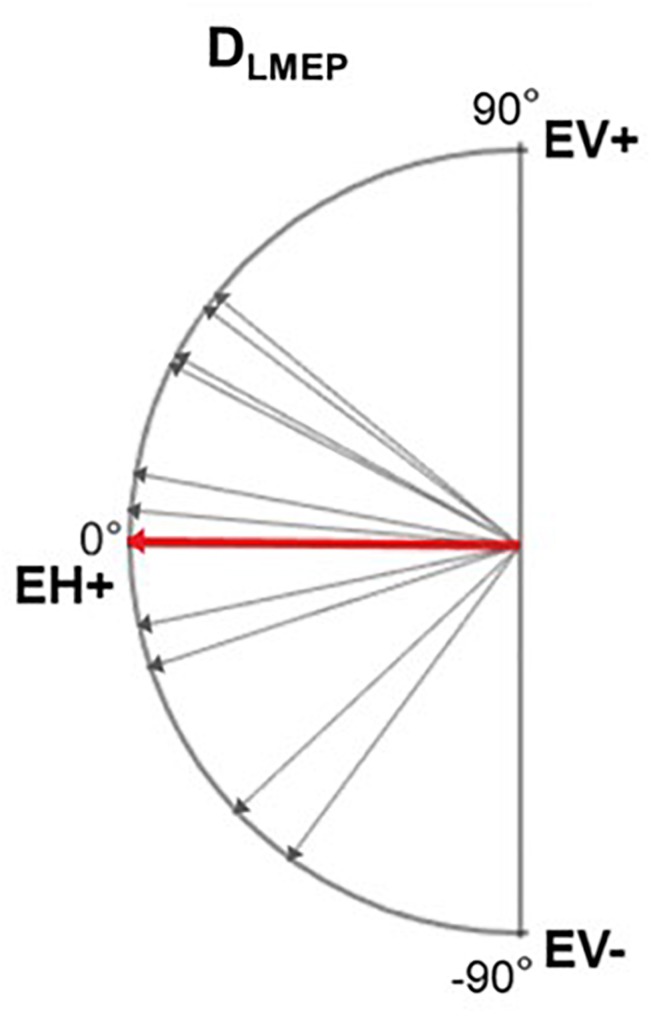
Electrical axis of LMEP dipole (D_LMEP_). A vector directed toward 0° means a horizontal vector directed to the right. A vector directed toward 90° means a vertical vector directed cranially. Single-patient (gray) and mean values (red) are displayed.

In 10 patients, LMEPs were recorded in both EH and EV channels and the LMEP dipole vector could be calculated (Figure 7). D_LMEP_ had a mean value of 0.51° (range, minimum −72.84° maximum 44.17). In all measurements dipoles were directed to the right side, if considering the horizontal plane (toward EH+) Table 5.

**TABLE 5:** Values of LMEP dipole electrical axis (D_LMEP_).

	**D_LMEP_(°)**
PAT 1	29.63
PAT 2	4.86
PAT 3	10.06
PAT 4	27.83
PAT 5	–11.36
PAT 6	40.56
PAT 7	–50.16
PAT 8	–72.84
PAT 9	44.17
PAT 10	–17.63
**Mean**	**0.51**

*A 0° angle equals to a horizontal axis directed to the right. Positive angles express a cranial deviation of the axis.*

### Electrode Placement

**FIGURE 8:**
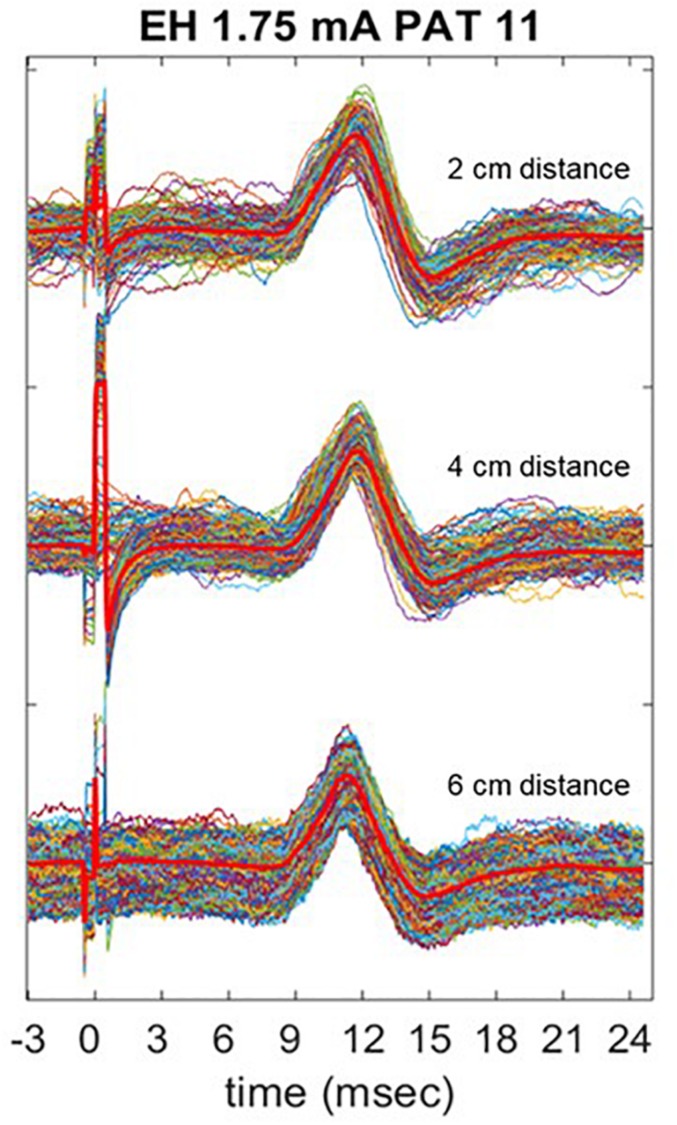
Comparison of LMEP recording with EH electrodes placed at 2, 4, and 6 cm laterally to the laryngeal prominence. The stimulation was performed at 1.75 mA of current output (Xstim). The bright red lines represent the averaged curve for each train. Muscular artifacts are more visible when 6 cm distance is used for the recording.

Figure 8 shows three trains of 600 pulses delivered at the same current output of 1.75 mA in one patient (PAT 11), recorded with EH electrodes placed at different distances in the horizontal plane. In Table 6, the mean P2P values, and CV are summarized. P2P values and CV are comparable across the three placements with no statistically significant difference. However, visual analysis of LMEP single epochs shows that high-frequency muscular artifacts particularly affect the recording with EH electrodes at 6 cm of distance (Figure 8).

**TABLE 6:** Results of LMEP peak-to-peak amplitude in PAT 11, as recorded with EH electrodes placed at 2, 4, and 6 cm laterally to the laryngeal prominence.

	**Mean P2P (μV)**	**SD (μV)**	**CV**
2 cm	191.58	25.73	13.43%
4 cm	171.89	22.65	13.18%
6 cm	172.21	25.65	14.89%

*P2P, peak-to-peak amplitude; SD, standard deviation; CV, coefficient of variation.*

## Discussion

This study shows that VNS-induced laryngeal muscular activation can be reliably recorded in a non-invasive manner, in patients chronically implanted with VNS. We successfully recorded LMEPs transcutaneously in 11 patients treated with VNS, at different time points during their therapy (from 8 months up to 14 years). The presence of LMEPs after many years of treatment with VNS therapy (>10 years) encourages their application in the chronic follow-up of the integrity of the lead-nerve interface. As valuable additional information to the electrode impedance test, measuring LMEPs can guarantee to the clinician an efficient activation of the nerve fibers.

The recorded LMEPs consisted of a N1 and P1 peak, with N1 earliest deflection appearing at 6.89 ms (range: 5.37–8.72 ms) after the first peak of the stimulation artifact. This finding is highly concordant with previous invasive assessments of VNS-induced laryngeal muscular responses reported by Ardesch et al. (2007), where a mean latency of 6.7 ms (range: 5.25–9.75 ms) was reported in eight patients. Thresholds for laryngeal muscle activation ranged between 0.25 and 1 mA, for a pulse width of 250 μs. With invasive recording and using the same stimulus, Ardesch et al. (2007) found a left vocal fold EMG-threshold between 0.25 and 0.50 mA, and saturation levels between 0.75 and 1.00 mA, which are comparable to our results.

The dipole orientation of the muscular response is mainly horizontal, directed to the right with a slight and variable inclination either cranially or caudally. The optimal placement of the surface recording electrodes is thus horizontal, and a single horizontal recording dipole is sufficient. We propose to place the electrodes symmetrically halfway between the laryngeal prominence and the medial edge of the sternocleidomastoid muscle. This corresponds to about 4 cm horizontally on each side of the laryngeal prominence (EH+/EH− derivation shown in Figure 1). As far as possible from muscular interferences, this placement allows to be anatomically as close as possible to the intrinsic laryngeal musculature, and inter-electrode distance of approximately 8 cm allows recording from deeper sources as required.

We found an excellent reliability (ICC > 0.9) of the 600 to 1200 responses within a single train, regardless of the amplitude metrics used. The high internal consistency not only highlights a noise level requiring little averaging but also indicates that the measurement considered is physiologically stable. The P2P and AUC metric are almost equally valuable, but in cases of partial demyelination for example, the P2P value might be reduced while the AUC remains constant. Both methods might thus have a complementary value in some cases. Moreover, our results also show that averaging a low number of epochs or in most cases single recordings is largely sufficient to study LMEP amplitude. This could be of interest in clinical routine, where time issues may be important. Only one patient (PAT 5), showed a lower SNR requiring 50 to 100 epochs to be gathered. Although breathing, swallowing or cardiac pulse-related artifacts may affect the SNR of LMEPs recordings, we demonstrated that by recording and averaging 50 to 100 epochs, a reliable quantification of the LMEPs can be provided in all cases. PAT 5 had been treated with VNS for 8 years, and did not correspond to the patient with the longest follow-up in our patient population (i.e., 14 years). We did not find significant differences in amplitude measurements of the LMEPs recorded from short and long-term follow-up patients, i.e., implanted for less than 4 years vs. more than 4 years (Englot et al., 2016).

Krahl et al. (2001) demonstrated that the effect of VNS on PTZ induced seizures is still present after selective lesion of the vagal C fibers with capsaicin, indicating that only low threshold A and B fibers are possible candidates for the beneficial effects of VNS. Many other studies further support the idea that low or medium stimulus intensities are sufficient to achieve the therapeutic effects (Zagon, 2000; Groves and Brown, 2005; Mollet et al., 2013). Although the conduction velocity in our study cannot be directly measured, due to a lack of information on the exact length of the recurrent laryngeal nerve in each patient, an estimate can be given. Taking into account the mean length of the recurrent laryngeal nerve in humans (Prades et al., 2012) and the average distance between placement of VNS electrode and branching of the nerve (Krahl, 2012), and subtracting the transmission time (1 ms) at the neuromuscular junction (Katz and Miledi, 1965) from our mean latency findings, we can estimate the conduction velocity to be 45.3 m/s (34.5–61.1 m/s). For this reason, we assume that the activated fibers are Aα-fibers corresponding to motor nerve fibers.

Until today, human data describing the effect of VNS on the vocal cords are mostly results of laryngological studies (Shaffer et al., 2005; Ardesch et al., 2007; Felisati et al., 2014; Al Omari et al., 2017; Saibene et al., 2017) sometimes combined with needle-EMG (Ardesch et al., 2007; Saibene et al., 2017). The main goal of these studies was to better understand the nature of voice disturbances in VNS-implanted patients. They pointed to the frequent and most often partial vagus nerve damage, typically temporary and recovering relatively quickly. However, the use of invasive and costly laryngological techniques is not realistic in the frame of a routine follow-up of epileptic patients treated with VNS. In contrast, the surface recording of LMEP could offer a solution. In our case, no test was performed earlier than 8 months after implantation, at a time where most nerve damages would have recovered. The use of LMEPs to monitor the nerve activation at an earlier, acute stage could be useful in guiding proper stimulus titration.

Our study mainly focused on feasibility and methodology of LMEPs recording, to be potentially used as biomarker of effective vagus nerve activation. A correlation of LMEPs and the clinical outcomes of the VNS therapy remained out of scope for this study. However, we expect that some poor clinical responses might be related to an impaired activation of the vagus nerve due to local gliosis, demyelination, or axonal loss. Such problems could result in absent LMEPs, reduced amplitudes, prolonged latencies, prolonged response durations, abnormal shapes, or distorted recruitment curves (Meulstee et al., 1997; Ridding and Rothwell, 1997). In our study population, 3/11 patients were considered responders to VNS (>50% seizure reduction). Interestingly, 2/3 of these patients (PAT2 and PAT 9) presented lower recruitment slope steepness and Boltzmann sigmoid curves could be fitted. Clearly, the stimulus strength resolution available to us was not sufficient to build a recruitment curve in all except 3 patients. As a strict minimum, we need at least one data point below threshold, one above saturation and two on the slope. Considering our results (Figure 6), a range of 0.15 to 1.5 mA with at least a 10 step logarithmic increment scale should be available in the future.

The three responders of our study population were responding at 1 mA of current output, and no increase in LMEPs amplitude was found in the following 1.25 mA train. Based on the link between fiber diameter and activation threshold, it is our expectation that the recruitment saturation of the clinically pertinent sensory afferent fibers is related to the saturation observed in motor fibers. The exact threshold ratio between motor fibers and therapeutically pertinent afferent fibers has still to be demonstrated. However, we found interesting that none of the investigated responder patients received stimulation currents stronger than the approximate 100% of the motor recruitment saturation, while in several non-responders the output current reaches the 200–250% of saturation.

Further studies are needed to elucidate the correlation between LMEP recruitment curves and: (i) clinical response, (ii) other markers of VNS-induced afferent activation (Hammond et al., 1992; Usami et al., 2013). These comparisons could validate the future use of LMEPs as a non-invasive read-out of therapeutic vagal fiber activation. The applied stimulation frequency in our study was 20 Hz, which is largely above the refractory period of the nerve (1 to 2 ms), making it possible to reliably study the amplitude behavior across patients and across trains of different current outputs. However, due to the reasons explained above, a more precise current output ideally of 0.0625–0.125 mA, would be necessary to quantify the recruitment in all patients.

Finally, hoping to limit any activation of the vagus nerve in the caudal direction, some authors have focused on the anodal block. However, the very existence of the LMEPs reported here proves that anodal block may, at most, have a negligible effect. Some solutions have been proposed to increase the anodal block effect by using depolarizing and slowly rising pulses (Vuckovic et al., 2008). This effect was mainly seen at very high charge densities, which do not apply to the clinical parameters of VNS. Moreover, Grimonprez et al. (2015) has shown in rats that with clinically relevant parameters, changing the polarity of the stimulation pulse did not affect the LMEP. Anodal block is therefore not an issue here.

Larger prospective studies are necessary to explore the full significance of this technique. Strength-duration curves could not be fitted in this study, due to technical programing constraints. However, considering the well-known relationship between threshold and nerve fiber diameter, a constant but as yet unknown ratio must exist between the threshold of the A-alpha motor fibers activated here and the threshold to activate the nerve fibers pertinent for the clinical effects of the VNS therapy (McAllen et al., 2018). A direct estimation of the necessary stimulus strength (current and duration) would then be possible on the basis of the LMEP threshold.

## Conclusion

In VNS-implanted patients, efferent activation of the recurrent laryngeal nerve leads to a local muscular activation recordable from the skin surface as LMEPs. The method only requires a single horizontal recording dipole using surface electrodes symmetrically placed on each side of the laryngeal prominence, at half the distance between the midline and the medial edge of the sternocleidomastoid muscle. LMEPs show very good reliability and measurability over a long period of time. At most, single trains of 50–100 stimuli at 20 Hz are sufficient to yield excellently consistent measures. LMEPs directly measure the effectiveness of electrical activation of motor efferent vagal nerve fibers. In the future, they could be used in a clinical context as a possible biomarker of effective nerve stimulation. Further studies are needed to assess the relation between LMEP characteristics and VNS clinical effect on seizures.

## Data Availability

The raw data supporting the conclusions of this manuscript will be made available by the authors, without undue reservation, to any qualified researcher.

## Author Contributions

SV, JD, and RET designed the research. SV, SFS, and RET assisted with patient recruitment. SV performed the neurophysiological recordings. SV and LS analyzed the data. SV, LS, CB, JD, HS, JC, AN, RR, KV, and RET interpreted the data. SV and RET wrote the manuscript. SV, LS, CB, JD, HS, JC, SFS, HR, AN, RR, KV, and RET contributed to the revision of the manuscript. All authors agreed to the content present in its final version.

## Conflict of Interest Statement

The authors declare that the research was conducted in the absence of any commercial or financial relationships that could be construed as a potential conflict of interest.

## References

[B1] Al Omari A. I. Alzoubi F. Q. Alsalem M. M. Aburahma S. K. Mardini D. T. Castellanos P. F. (2017). The vagal nerve stimulation outcome, and laryngeal effect: otolaryngologists roles and perspective. *Am. J. Otolaryngol.* 38 408–413. 10.1016/j.amjoto.2017.03.011 28390806

[B2] Ardesch J. J. Buschman H. P. J. Wagener-Schimmel L. J. J. C. van der Aa H. E. Hageman G. (2007). Vagus nerve stimulation for medically refractory epilepsy: a long-term follow-up study. *Seizure* 16 579–585. 10.1016/j.seizure.2007.04.005 17543546

[B3] Ardesch J. J. Sikken J. R. Veltink P. H. van der Aa H. E. Hageman G. Buschman H. P. J. (2010). Vagus nerve stimulation for epilepsy activates the vocal folds maximally at therapeutic levels. *Epilepsy Res.* 89 227–231. 10.1016/j.eplepsyres.2010.01.005 20129758

[B4] Ben-Menachem E. Manon-Espaillat R. Ristanovic R. Wilder B. J. Stefan H. Mirza W. (1994). Vagus nerve stimulation for treatment of partial seizures: 1. A controlled study of effect on seizures. First international vagus nerve stimulation study group. *Epilepsia* 35 616–626. 10.1111/j.1528-1157.1994.tb02482.x 8026408

[B5] Boon P. Vonck K. de Reuck J. Caemaert J. (2002). Vagus nerve stimulation for refractory epilepsy. *Seizure* 11(Suppl. A), 448–455. 10.1053/seiz.2001.0626 12185767

[B6] Bouckaert C. Raedt R. Gadeyne S. Carrette E. Proesmans S. Dewaele F. (2018). Laryngeal motor-evoked potentials as an indicator of vagus nerve activation. *Eur. J. Neurol.* 25:45.

[B7] Cacchio A. Cimini N. Alosi P. Santilli V. Marrelli A. (2009). Reliability of transcranial magnetic stimulation-related measurements of tibialis anterior muscle in healthy subjects. *Clin. Neurophysiol.* 120 414–419. 10.1016/j.clinph.2008.11.019 19135412

[B8] Cohen J. Polich J. (1997). On the number of trials needed for P300. *Int. J. Psychophysiol.* 25 249–255. 10.1016/s0167-8760(96)00743-x 9105949

[B9] Cronbach L. J. Warrington W. G. (1951). Time-limit tests: estimating their reliability and degree of speeding. *Psychometrika* 16 167–188. 10.1007/bf02289113 14844557

[B10] DeGiorgio C. M. Schachter S. C. Handforth A. Salinsky M. Thompson J. Uthman B. (2000). Prospective long-term study of vagus nerve stimulation for the treatment of refractory seizures. *Epilepsia* 41 1195–1200. 10999559 10.1111/j.1528-1157.2000.tb00325.x

[B11] Devanne H. Lavoie B. A. Capaday C. (1997). Input-output properties and gain changes in the human corticospinal pathway. *Exp. Brain Res.* 114 329–338. 10.1007/pl00005641 9166922

[B12] El Tahry R. Mollet L. Raedt R. Delbeke J. De Herdt V. Wyckhuys T. (2011). Repeated assessment of larynx compound muscle action potentials using a self-sizing cuff electrode around the vagus nerve in experimental rats. *J. Neurosci. Methods* 198 287–293. 10.1016/j.jneumeth.2011.04.007 21513735

[B13] Englot D. J. Chang E. F. Auguste K. I. (2011). Vagus nerve stimulation for epilepsy: a meta-analysis of efficacy and predictors of response. *J. Neurosurg.* 115 1248–1255. 10.3171/2011.7.JNS11977 21838505

[B14] Englot D. J. Rolston J. D. Wright C. W. Hassnain K. H. Chang E. F. (2016). Rates and predictors of seizure freedom with vagus nerve stimulation for intractable epilepsy. *Neurosurgery* 79 345–353. 10.1227/NEU.0000000000001165 26645965 PMC4884552

[B15] Fabiani M. Gratton G. Karis D. Donchin E. (1987). Definition, identification, and reliability of measurement of the P300 component of the event-related brain potential. *Adv. Psychophysiol.* 2:78.

[B16] Felisati G. Gardella E. Schiavo P. Saibene A. M. Pipolo C. Bertazzoli M. (2014). Endoscopic laryngeal patterns in vagus nerve stimulation therapy for drug-resistant epilepsy. *Eur. Arch. Otorhinolaryngol.* 271 117–123. 10.1007/s00405-013-2568-z 23744179

[B17] Ghaemi K. Elsharkawy A. E. Schulz R. Hoppe M. Polster T. Pannek H. (2010). Vagus nerve stimulation: outcome and predictors of seizure freedom in long-term follow-up. *Seizure* 19 264–268. 10.1016/j.seizure.2010.03.002 20362466

[B18] Goldsworthy M. R. Hordacre B. Ridding M. C. (2016). Minimum number of trials required for within- and between-session reliability of TMS measures of corticospinal excitability. *Neuroscience* 320 205–209. 10.1016/j.neuroscience.2016.02.012 26872998

[B19] Green S. B. Yang Y. Alt M. Brinkley S. Gray S. Hogan T. (2016). Use of internal consistency coefficients for estimating reliability of experimental task scores. *Psychon. Bull. Rev.* 23 750–763. 10.3758/s13423-015-0968-963 26546100 PMC5484005

[B20] Grimonprez A. Raedt R. De Taeye L. Larsen L. E. Delbeke J. Boon P. (2015). A preclinical study of laryngeal motor-evoked potentials as a marker vagus nerve activation. *Int. J. Neural Syst.* 25:1550034. 10.1142/S0129065715500343 26510476

[B21] Groves D. A. Brown V. J. (2005). Vagal nerve stimulation: a review of its applications and potential mechanisms that mediate its clinical effects. *Neurosci. Biobehav. Rev.* 29 493–500. 10.1016/j.neubiorev.2005.01.004 15820552

[B22] Hammond E. J. Uthman B. M. Reid S. A. Wilder B. J. (1992). Electrophysiologic studies of cervical vagus nerve stimulation in humans: II. Evoked potentials. *Epilepsia* 33 1021–1028. 10.1111/j.1528-1157.1992.tb01753.x 1464258

[B23] Handforth A. DeGiorgio C. M. Schachter S. C. Uthman B. M. Naritoku D. K. Tecoma E. S. (1998). Vagus nerve stimulation therapy for partial-onset seizures: a randomized active-control trial. *Neurology* 51 48–55. 10.1212/wnl.51.1.48 9674777

[B24] Hinton P. R. McMurray I. Brownlow C. (2014). *SPSS Explained.* Abingdon: Routledge.

[B25] Joyeux L. Deprez M. Khatoun A. Van Kuyck K. Pelsmaekers K. Engels A. C. (2017). Quantitative analysis of motor evoked potentials in the neonatal lamb. *Sci. Rep.* 7:16095. 10.1038/s41598-017-16453-16458 29170524 PMC5701025

[B26] Katz B. Miledi R. (1965). The effect of temperature on the synaptic delay at the neuromuscular junction. *J. Physiol.* 181 656–670. 10.1113/jphysiol.1965.sp007790 5880384 PMC1357674

[B27] Kersing W. Dejonckere P. H. van der Aa H. E. Buschman H. P. J. (2002). Laryngeal and vocal changes during vagus nerve stimulation in epileptic patients. *J. Voice* 16 251–257. 10.1016/s0892-1997(02)00094-2 12150377

[B28] Koo T. K. Li M. Y. (2016). A guideline of selecting and reporting intraclass correlation coefficients for reliability research. *J. Chiropr. Med.* 15 155–163. 10.1016/j.jcm.2016.02.012 27330520 PMC4913118

[B29] Krahl S. E. (2012). Vagus nerve stimulation for epilepsy: a review of the peripheral mechanisms. *Surg. Neurol. Int.* 3 S47–S52. 10.4103/2152-7806.91610 22826811 PMC3400480

[B30] Krahl S. E. Clark K. B. (2012). Vagus nerve stimulation for epilepsy: a review of central mechanisms. *Surg. Neurol. Int.* 3 S255–S259. 10.4103/2152-7806.103015 23230530 PMC3514919

[B31] Krahl S. E. Senanayake S. S. Handforth A. (2001). Destruction of peripheral C-fibers does not alter subsequent vagus nerve stimulation-induced seizure suppression in rats. *Epilepsia* 42 586–589. 10.1046/j.1528-1157.2001.09700.x 11380564

[B32] Kwan P. Arzimanoglou A. Berg A. T. Brodie M. J. Allen Hauser W. Mathern G. (2010). Definition of drug resistant epilepsy: consensus proposal by the ad hoc task force of the ILAE commission on therapeutic strategies. *Epilepsia* 51 1069–1077. 10.1111/j.1528-1167.2009.02397.x 19889013

[B33] Lavoie B. A. Cody F. W. Capaday C. (1995). Cortical control of human soleus muscle during volitional and postural activities studied using focal magnetic stimulation. *Exp. Brain Res.* 103 97–107. 7615042 10.1007/BF00241968

[B34] McAllen R. M. Shafton A. D. Bratton B. O. Trevaks D. Furness J. B. (2018). Calibration of thresholds for functional engagement of vagal A, B and C fiber groups in vivo. *Bioelectron. Med.* 1 21–27. 10.2217/bem-2017-0001 29480903 PMC5811083

[B35] McGraw K. O. Wong S. P. (1996). Forming inferences about some intraclass correlation coefficients. *Psychol. Methods* 1 30–46. 10.1037//1082-989x.1.1.30

[B36] Meulstee J. Darbas A. van Doorn P. A. van Briemen L. van der Meche F. G. (1997). Decreased electrical excitability of peripheral nerves in demyelinating polyneuropathies. *J. Neurol. Neurosurg. Psychiatry* 62 398–400. 10.1136/jnnp.62.4.398 9120460 PMC1074103

[B37] Mollet L. Raedt R. Delbeke J. El Tahry R. Grimonprez A. Dauwe I. (2013). Electrophysiological responses from vagus nerve stimulation in rats. *Int. J. Neural Syst.* 23:1350027. 10.1142/S0129065713500275 24156670

[B38] Okamoto E. Ishikawa E. Yamamoto T. Matsuda M. Nakai K. Matsushita A. (2015). Variability in amplitude and stimulation threshold values in motor evoked potential (MEP) monitoring during the resection of brain lesions. *Clin. Neurophysiol.* 126 1271–1278. 10.1016/j.clinph.2014.10.005 25454280

[B39] Olvet D. M. Hajcak G. (2009). Reliability of error-related brain activity. *Brain Res.* 1284 89–99. 10.1016/j.brainres.2009.05.079 19501071

[B40] Prades J. M. Dubois M. D. Dumollard J. M. Tordella L. Rigail J. Timoshenko A. P. (2012). Morphological and functional asymmetry of the human recurrent laryngeal nerve. *Surg. Radiol. Anat.* 34 903–908. 10.1007/s00276-012-0999-997 23150169

[B41] Ridding M. C. Rothwell J. C. (1997). Stimulus/response curves as a method of measuring motor cortical excitability in man. *Electroencephalogr. Clin. Neurophysiol.* 105 340–344. 10.1016/s0924-980x(97)00041-6 9362997

[B42] Rietdijk W. J. R. Franken I. H. A. Thurik A. R. (2014). Internal consistency of event-related potentials associated with cognitive control: N2/P3 and ERN/Pe. *PLoS One* 9:e102672. 10.1371/journal.pone.0102672 25033272 PMC4102542

[B43] Saibene A. M. Zambrelli E. Pipolo C. Maccari A. Felisati G. Felisati E. (2017). The role of laryngeal electromyography in vagus nerve stimulation-related vocal fold dysmotility. *Eur. Arch. Otorhinolaryngol.* 274 1585–1589. 10.1007/s00405-016-4344-4343 27738822

[B44] Shaffer M. J. Jackson C. E. Szabo C. A. Simpson C. B. (2005). Vagal nerve stimulation: clinical and electrophysiological effects on vocal fold function. *Ann. Otol. Rhinol. Laryngol.* 114 7–14. 10.1177/000348940511400103 15697156

[B45] Thigpen N. N. Kappenman E. S. Keil A. (2017). Assessing the internal consistency of the event-related potential: an example analysis. *Psychophysiology* 54 123–138. 10.1111/psyp.12629 28000264 PMC5525326

[B46] Usami K. Kawai K. Sonoo M. Saito N. (2013). Scalp-recorded evoked potentials as a marker for afferent nerve impulse in clinical vagus nerve stimulation. *Brain Stimul.* 6 615–623. 10.1016/j.brs.2012.09.007 23088852

[B47] Vonck K. Thadani V. Gilbert K. Dedeurwaerdere S. De Groote L. De Herdt V. (2004). Vagus nerve stimulation for refractory epilepsy: a transatlantic experience. *J. Clin. Neurophysiol.* 21 283–289. 15509917 10.1097/01.wnp.0000139654.32974.4e

[B48] Vuckovic A. Tosato M. Struijk J. J. (2008). A comparative study of three techniques for diameter selective fiber activation in the vagal nerve: anodal block, depolarizing prepulses and slowly rising pulses. *J. Neural Eng.* 5 275–286. 10.1088/1741-2560/5/3/002 18566504

[B49] Zagon A. (2000). Activation of cardiac vagal afferents facilitates late vagal inhibition in neurones of the rostral ventrolateral medulla oblongata bilaterally. *Brain Res.* 854 172–177. 10.1016/s0006-8993(99)02338-0 10784119

[B50] Zalvan C. Sulica L. Wolf S. Cohen J. Gonzalez-Yanes O. Blitzer A. (2003). Laryngopharyngeal dysfunction from the implant vagal nerve stimulator. *Laryngoscope* 113 221–225. 10.1097/00005537-200302000-200302005 12567072

